# The paraventricular nucleus of the thalamus is recruited by both natural rewards and drugs of abuse: recent evidence of a pivotal role for orexin/hypocretin signaling in this thalamic nucleus in drug-seeking behavior

**DOI:** 10.3389/fnbeh.2014.00117

**Published:** 2014-04-03

**Authors:** Alessandra Matzeu, Eva R. Zamora-Martinez, Rémi Martin-Fardon

**Affiliations:** Molecular and Cellular Neuroscience Department, The Scripps Research InstituteLa Jolla, CA, USA

**Keywords:** paraventricular nucleus of the thalamus, orexin/hypocretin, drug addiction, drug-seeking behavior, natural reward

## Abstract

A major challenge for the successful treatment of drug addiction is the long-lasting susceptibility to relapse and multiple processes that have been implicated in the compulsion to resume drug intake during abstinence. Recently, the orexin/hypocretin (Orx/Hcrt) system has been shown to play a role in drug-seeking behavior. The Orx/Hcrt system regulates a wide range of physiological processes, including feeding, energy metabolism, and arousal. It has also been shown to be recruited by drugs of abuse. Orx/Hcrt neurons are predominantly located in the lateral hypothalamus that projects to the paraventricular nucleus of the thalamus (PVT), a region that has been identified as a “way-station” that processes information and then modulates the mesolimbic reward and extrahypothalamic stress systems. Although not thought to be part of the “drug addiction circuitry”, recent evidence indicates that the PVT is involved in the modulation of reward function in general and drug-directed behavior in particular. Evidence indicates a role for Orx/Hcrt transmission in the PVT in the modulation of reward function in general and drug-directed behavior in particular. One hypothesis is that following repeated drug exposure, the Orx/Hcrt system acquires a preferential role in mediating the effects of drugs *vs*. natural rewards. The present review discusses recent findings that suggest maladaptive recruitment of the PVT by drugs of abuse, specifically Orx/Hcrt-PVT neurotransmission.

## Introduction

Drug addiction is a chronic relapsing disorder characterized by persistent drug-seeking and drug-taking behaviors (O’Brien and McLellan, 1996; Leshner, 1997; O’Brien et al., 1998; McLellan et al., 2000). Elucidation of the neurobiological mechanisms that underlie the chronically relapsing nature of addiction and identification of pharmacological treatment targets for relapse prevention has emerged as a central issue in addiction research.

Several studies have sought to clarify the neuronal substrates that regulate the compulsive behavioral characteristics of addiction. Brain regions that have been identified to be involved in relapse (drug seeking)-like behavior include the medial prefrontal cortex, basolateral amygdala, central nucleus of the amygdala, bed nucleus of the stria terminalis, hippocampus, nucleus accumbens, and dorsal striatum (Everitt et al., 2001; McFarland and Kalivas, 2001; Cardinal et al., 2002; Goldstein and Volkow, 2002; Ito et al., 2002; See, 2002; Kalivas and Volkow, 2005; Weiss, 2005; Belin and Everitt, 2008; Steketee and Kalivas, 2011). Recently, emerging evidence has proposed that the thalamus could also be included in the neurocircuitry of addiction. Indeed, it is considered an important key relay between the ventral striatopallidum and dorsal striatum and may contribute to the development of compulsive drug-seeking behavior (Pierce and Vanderschuren, 2010).

Among the nuclei of the thalamus, the paraventricular nucleus of the thalamus (PVT) has a pivotal neuroanatomical position and therefore influences structures that have been implicated in drug-seeking behavior (Moga et al., 1995; Bubser and Deutch, 1998; Van der Werf et al., 2002). Of notable relevance for this review is hypothalamic orexin/hypocretin (Orx/Hcrt) innervation of the PVT. Orx/Hcrt peptides are found in fibers located in all regions of this thalamic nucleus, whereas relatively modest fiber density is found in the adjacent midline and intralaminar thalamic nuclei (Kirouac et al., 2005). Although compelling evidence shows a role for Orx/Hcrt in arousal and maintenance of the waking state (de Lecea, 2012), further evidence supports an important and specific role in general reward processing and drug abuse in particular (for review, see Mahler et al., 2012).

An important consideration when referring to general reward processing is what differentiates neural signaling related to “normal” appetitive behavior *vs*. drug-directed behavior. One possibility is that the neuronal circuits that mediate the control of drug-seeking and drug-taking behaviors are common motivational neuronal substrates that are more robustly activated by drugs and are not specific for addiction-related processes. Drug-induced neuronal activation that “normally” controls responding for natural rewards could create new motivational states or redirect signaling that normally controls responses for natural reward toward drug-directed behavior (Kelley and Berridge, 2002). The aim of this review is to summarize recent findings that suggest maladaptive recruitment of the PVT by drugs of abuse, specifically Orx/Hcrt-PVT transmission, as a new neurotransmission system in the etiology of compulsive drug seeking.

## The PVT

The PVT lies adjacent to the dorsal aspect of the third ventricle. The PVT is part of dorsal midline thalamic nuclei and plays a significant role in functions related to arousal, attention, and awareness (Bentivoglio et al., 1991; Groenewegen and Berendse, 1994; Van der Werf et al., 2002). Although the midline and intralaminar thalamic nuclei were first hypothesized to participate in the processing of non-discriminative nociceptive inputs (Berendse and Groenewegen, 1991), it is now well recognized that each member of these nuclei innervates functionally distinct areas of the cortex and striatum (Groenewegen and Berendse, 1994; Van der Werf et al., 2002; Smith et al., 2004).

Neuroanatomical studies have shown that the PVT receives projections from brainstem regions associated with arousal and autonomic nervous system function (Cornwall and Phillipson, 1988b; Chen and Su, 1990; Ruggiero et al., 1998; Krout and Loewy, 2000; Krout et al., 2002; Hsu and Price, 2009). Furthermore, the PVT, through its projections to the prefrontal cortex and nucleus accumbens (Berendse and Groenewegen, 1990; Su and Bentivoglio, 1990; Brog et al., 1993; Freedman and Cassell, 1994; Moga et al., 1995; Bubser and Deutch, 1998; Otake and Nakamura, 1998; Parsons et al., 2007; Li and Kirouac, 2008; Vertes and Hoover, 2008; Hsu and Price, 2009), places this thalamic structure in a unique position to affect cortico-striatal mechanisms involved in reward and motivation (Pennartz et al., 1994; Cardinal et al., 2002; Walker et al., 2003).

The PVT receives large and distinct inputs from several areas of the hypothalamus, including the suprachiasmatic, arcuate, dorsomedial, and ventromedial nuclei, and preoptic and lateral hypothalamic areas (Cornwall and Phillipson, 1988a; Chen and Su, 1990; Novak et al., 2000a; Peng and Bentivoglio, 2004; Kirouac et al., 2005, 2006; Otake, 2005; Hsu and Price, 2009), critical structures for the expression of motivated behavior (Swanson, 2000). Remarkably, the PVT is the target of Orx/Hcrt hypothalamic neurons (Kirouac et al., 2005) and has been shown to function as an interface between the hypothalamus and cortical-striatal projections that are essential for the integration of energy balance, arousal, and food reward (e.g., Kelley et al., 2005).

Experiments that investigated neuronal activation of the PVT have consistently shown that this brain region is recruited during periods of arousal or by stress (Peng et al., 1995; Bhatnagar and Dallman, 1998; Novak and Nunez, 1998; Bubser and Deutch, 1999; Novak et al., 2000b; Otake et al., 2002). The PVT has also been implicated in the regulation of food intake and hypothalamic-pituitary-adrenal activity in response to chronic stress, food consumption, and energy balance (Bhatnagar and Dallman, 1998, 1999; Jaferi et al., 2003). Although not initially included in the neurocircuitry of addiction, recent evidence implicates the PVT in the modulation of drug-directed behavior. In fact, the PVT projects to brain regions that are implicated in the control of drug-seeking behavior, such as the nucleus accumbens, amygdala, bed nucleus of the stria terminalis, and prefrontal cortex (Moga et al., 1995; Bubser and Deutch, 1998; Van der Werf et al., 2002). Importantly, earlier findings demonstrated selective activation of the PVT during ethanol seeking (Dayas et al., 2008; Hamlin et al., 2009), and recent evidence has shown potent and selective activation of the PVT during cocaine seeking that does not occur during natural reward (e.g., a highly palatable conventional reinforcer) seeking (Martin-Fardon et al., 2013). Among the several functions mentioned above, this review discusses the involvement of the PVT in drug- *vs*. natural reward-seeking behavior (a nondrug control). This review uses the terms “conventional reinforcer” or “natural reward” to loosely define a nondrug condition (usually a sweet highly palatable solution) that will serve as a comparison control for the drug.

## The Orx/Hcrt system

Orx/Hcrt peptides, orexin A and B (Orx-A and Orx-B), also known as hypocretins (Hcrt-1 and Hcrt-2), are neuropeptides expressed exclusively in neurons of dorsal tuberal hypothalamic nuclei: lateral hypothalamus, perifornical nucleus, and dorsomedial hypothalamus (de Lecea et al., 1998; Sakurai et al., 1998b). Orx-A/Hcrt-1 and Orx-B/Hcrt-2 are products of a common single precursor polypeptide, prepro-orexin, through usual proteolytic processing (de Lecea et al., 1998). These peptides share sequence similarity and are the ligands for two receptors: Hcrt-r1 and Hcrt-r2. Hcrt-r1 binds Orx-A with 20–30 nM affinity but has much lower affinity (10- to 1000-fold lower) for Orx-B, whereas Hcrt-r2 binds both peptides with similar affinity (in the 40 nM range; Sakurai et al., 1998a; Ammoun et al., 2003; Scammell and Winrow, 2011). Many studies have suggested that Orx/Hcrt receptors are coupled to G-proteins. However, the G-coupling of these receptors is far from clear but based on several findings both Hcrt-r1 and Hcrt-r2 are likely to couple G_i/o_, G_s_ and G_q_ family G-proteins (Gotter et al., 2012; Kukkonen, 2013).

Orx/Hcrt neurons receive inputs from numerous brain areas and project to the entire brain, thus influencing multiple neuronal circuitries (Peyron et al., 1998; Date et al., 1999; Nambu et al., 1999). Dense Orx/Hcrt terminals can be found in the cerebral cortex, olfactory bulb, hippocampus, amygdala, basal forebrain, hypothalamus, tuberomammillary nucleus, PVT, arcuate nucleus of the hypothalamus, and brainstem (Peyron et al., 1998; Date et al., 1999; Nambu et al., 1999). Orx/Hcrt neurons receive projections from the medial prefrontal cortex, nucleus accumbens shell, amygdala, bed nucleus of the stria terminalis, arcuate nucleus of the hypothalamus, and preoptic area (Sakurai et al., 2005). With regard to Hcrt-rs, limited overlapping distributions of Hcrt-r1 and Hcrt-r2 mRNAs have been shown, with functional differences between Hcrt-r1 and Hcrt-r2 (Trivedi et al., 1998; Lu et al., 2000; Marcus et al., 2001; for review, see Aston-Jones et al., 2010), proposing different physiological roles for each receptor subtype.

Because of its connections, the Orx/Hcrt system is involved in a multitude of physiological functions. The Orx/Hcrt system is strongly involved in the regulation of feeding, arousal, sleep/wake states, the stress response, energy homeostasis, and reward (for review, see Tsujino and Sakurai, 2013). Particularly important for this review, evidence supports an important and specific role for the Orx/Hcrt system in drug addiction (for review, see Mahler et al., 2012), specifically Orx/Hcrt neurons located in the lateral hypothalamus (Harris et al., 2005). Notably, these neurons project to the PVT, nucleus accumbens shell, ventral pallidum, ventral tegmental area, central nucleus of the amygdala, and bed nucleus of the stria terminalis (Peyron et al., 1998; Baldo et al., 2003; Winsky-Sommerer et al., 2004). Originally implicated in the regulation of feeding behavior (Sakurai et al., 1998a; Edwards et al., 1999; Haynes et al., 2000, 2002), these neurons play a modulatory role in reward function, with a specific contribution to drug-related behavior (Harris et al., 2005).

## The Orx/Hcrt system contibutes to the behavioral effects of drugs of abuse

Orx/Hcrt has been reported to enhance the incentive motivational effects of stimuli conditioned to drug availability, increase the motivation to seek the drug, and increase the reinforcing actions of drugs of abuse.

In fact, intra-ventral tegmental area microinjection of Orx-A produces a renewal of morphine-induced conditioned place preference (CPP), whereas administration of the Hcrt-r1 antagonist *N*-(2-methyl-6-benzoxazolyl)-*N*′-1,5-n-aphthyridin-4-yl urea (SB334867) attenuates the expression of morphine-induced CPP (Harris et al., 2005). Consistent with the role of Orx/Hcrt in the expression of CPP, when injected systematically, the Hcrt-r1 antagonists SB334867 and 5-bromo-*N*-[(2*S*,5*S*)-1-(3-fluoro-2-methoxybenzoyl)-5-methylpiperidin-2-yl]methyl-pyridin-2-amine (GSK1059865) reduce the expression of cocaine- and amphetamine-induced CPP (Gozzi et al., 2011; Hutcheson et al., 2011; Sartor and Aston-Jones, 2012), suggesting a prominent role for Hcrt-r1 in the rewarding effects of cocaine and amphetamine. Interestingly, the participation of Hcrt-r2 was recently described in some of ethanol’s behavioral effects. Blockade of Hcrt-r2 using (2,4-dibromo-phenyl)-3-([4*S*,5*S*]-2,2-dimethyl-4-phenyl-[1,3]dioxan-5-yl)-urea (JNJ-10397049) was reported to decrease the acquisition, expression, and reinstatement of ethanol-induced CPP (Shoblock et al., 2011), suggesting that Hcrt-r2 may be mainly involved in the rewarding effect of ethanol.

Orx/Hcrt has also been described to play a role in psychostimulant-induced locomotor sensitization. SB334867 injected peripherally or into the ventral tegmental area blocked the acquisition of cocaine sensitization, antagonized the potentiation of excitatory currents induced by cocaine in dopaminergic neurons of the ventral tegmental area (Borgland et al., 2006), and blocked the expression of amphetamine sensitization (Quarta et al., 2010). Moreover the dual Hcrt-r1/Hcrt-r-2 antagonist N-biphenyl-2-yl-1-[[(1-methyl-1H-benzimidazol-2-yl)sulfanyl]acetyl]-l-prolinamide similarly blocked the expression of amphetamine sensitization and plasticity-related gene expression in the ventral tegmental area after chronic amphetamine (Winrow et al., 2010).

Orx/Hcrt has also been reported to participate in regulating the motivation to take drugs. When injected in the ventral tegmental area, Orx-A/Hcrt-1 increases the breakpoint for cocaine self-administration on a progressive-ratio schedule of reinforcement (España et al., 2011). Antagonizing Hcrt-r1 with SB334867 reduces the motivation to self-administer cocaine and attenuates the cocaine-induced enhancement of dopaminergic signaling in the nucleus accumbens when injected into the ventral tegmental area (España et al., 2010). Additionally, the blockade of Hcrt-r1 decreases nicotine (Hollander et al., 2008) and heroin (Smith and Aston-Jones, 2012) self-administration, and both Hcrt-r1 or Hcrt-r2 antagonism reduces ethanol self-administration, without interfering with sucrose self-administration (Lawrence et al., 2006; Shoblock et al., 2011; Brown et al., 2013). Finally, recent findings have shown that Hcrt-r2 antagonism reduces compulsive heroin self-administration (Schmeichel et al., 2013).

Orx/Hcrt plays an important role in drug-seeking behavior triggered by stress or drug-related environmental stimuli. Intracerebroventricular (ICV) injection of Orx-A/Hcrt-1 increases intracranial self-stimulation (ICSS) thresholds and reinstates cocaine and nicotine seeking (Boutrel et al., 2005; Plaza-Zabala et al., 2010). Furthermore, the blockade of Hcrt-r1 prevents cue- and stress-induced reinstatement of cocaine, ethanol, and heroin seeking (Boutrel et al., 2005; Lawrence et al., 2006; Richards et al., 2008; Smith et al., 2010; Jupp et al., 2011b; Smith and Aston-Jones, 2012; Martin-Fardon and Weiss, 2014a,b).

The Orx/Hcrt system has also been shown to play a role in drug withdrawal. SB334867 attenuates the somatic signs of nicotine and morphine withdrawal (Sharf et al., 2008; Plaza-Zabala et al., 2012), and Orx/Hcrt neurons are activated following acute nicotine administration and during nicotine (Pasumarthi et al., 2006; Plaza-Zabala et al., 2012) and morphine (Georgescu et al., 2003) withdrawal. Some studies suggest the existence of a correlation between blood Orx/Hcrt levels and the symptoms of withdrawal from alcohol in humans (Bayerlein et al., 2011; von der Goltz et al., 2011), supporting the hypothesis that the Orx/Hcrt system is important for behavioral changes associated with drug dependence and withdrawal in animals and humans.

A central role for Orx/Hcrt neurons in the lateral hypothalamus in drug addiction exists (Harris et al., 2005). Orx/Hcrt neurons in the lateral hypothalamus become activated by stimuli associated with cocaine, ethanol, morphine, and food (Harris et al., 2005; Dayas et al., 2008; Martin-Fardon et al., 2010; Jupp et al., 2011b), and Orx/Hcrt microinjection in the lateral hypothalamus increases voluntary ethanol intake (Schneider et al., 2007). The expression of CPP induced by food, morphine, and cocaine is associated with the activation of lateral hypothalamus Orx/Hcrt neurons (Harris et al., 2005). Interestingly, cocaine-induced CPP was associated with a decrease in Orx/Hcrt mRNA expression in the lateral hypothalamus, suggesting some form of compensatory feedback that follows strong neuronal activation induced by cocaine (Zhou et al., 2008).

Behavioral and functional evidence indicates a role for Orx/Hcrt signaling in the neurobehavioral and motivational effects of ethanol and other drugs of abuse (Borgland et al., 2006; Bonci and Borgland, 2009; Thompson and Borgland, 2011). Importantly, Orx/Hcrt are hypothalamic neuropeptides that were originally reported to regulate feeding (Sakurai et al., 1998a). The blockade of Hcrt-r1 by SB334867 decreases food intake (Haynes et al., 2000; Rodgers et al., 2001; Ishii et al., 2005), and the Orx/Hcrt system appears to be recruited in regulating the intake of highly palatable food (Nair et al., 2008; Borgland et al., 2009; Choi et al., 2010).

Although the Orx/Hcrt system is well known to regulate (natural) reward function, the findings mentioned above indicate that the Orx/Hcrt system also plays a critical role in the neurobehavioral and motivational effects of drugs of abuse. Recent studies indicated that the Orx/Hcrt system is, in fact, more strongly engaged by drugs of abuse than by non-drug reinforcers. For example, Hcrt-r1 or Hcrt-r2 blockade is more effective in reducing ethanol self-administration than sucrose intake (Shoblock et al., 2011; Jupp et al., 2011a; Brown et al., 2013). Additionally, using a conditioned reinstatement animal model of relapse, in which stimuli conditioned to cocaine, ethanol, and conventional reinforcers elicit equal levels of reinstatement, pharmacological manipulation of Hcrt-r1 selectively reversed conditioned reinstatement induced by a cocaine- or ethanol-related stimulus but had no effects on the same stimulus conditioned to a conventional reinforcer (Martin-Fardon and Weiss, 2009, 2014a,b; Martin-Fardon et al., 2010).

## The PVT contributes to drug-seeking behavior

The PVT has been proposed to be a key relay that gates Orx/Hcrt-coded reward-related communication between the lateral hypothalamus and ventral and dorsal striatum (Kelley et al., 2005). This hypothalamic-thalamic-striatal neurocircuitry may have evolved to prolong central motivational states and promote feeding beyond the fulfillment of immediate energy needs, thereby creating energy reserves for potential future food shortages (Kelley et al., 2005). It is hypothesized that maladaptive recruitment of this system by drugs of abuse may “tilt” its function toward excessive drug-directed behavior, which may explain the increased sensitivity of the Orx/Hcrt system to antagonist interference with drug-seeking behavior as opposed to behavior directed toward natural reward.

Much evidence supports the involvement of the PVT in the reinstatement of drug-seeking behavior especially triggered by stimuli conditioned to the availability of the drug itself. For example, context- or cue-induced reinstatement of alcohol seeking is associated with significant PVT recruitment (Wedzony et al., 2003; Dayas et al., 2008; Perry and McNally, 2013). Furthermore, inactivation of the PVT prevents context-induced reinstatement of ethanol seeking (Hamlin et al., 2009; Marchant et al., 2010), cocaine prime-induced reinstatement (James et al., 2010), cocaine sensitization (Young and Deutch, 1998), and the expression of cocaine-induced CPP (Browning et al., 2014). Moreover, PVT neurons are activated by reexposure to cocaine-paired (Brown et al., 1992; Franklin and Druhan, 2000), methamphetamine-paired (Rhodes et al., 2005), and ethanol-paired (Wedzony et al., 2003; Dayas et al., 2008) contextual stimuli, whereas exposure to sucrose-related stimuli does not induce PVT activation (Wedzony et al., 2003).

In addition to the numerous studies that showed a contribution of the PVT in different aspects of drug addiction, the specific contribution of Orx/Hcrt signaling in this thalamic nucleus has recently attracted much attention. The PVT is densely innervated by Orx/Hcrt fibers (Kirouac et al., 2005; Parsons et al., 2006) and is a major source of glutamatergic afferents to the nucleus accumbens, bed nucleus of the stria terminalis, central nucleus of the amygdala, and medial prefrontal cortex (Parsons et al., 2007; Li and Kirouac, 2008; Vertes and Hoover, 2008; Hsu and Price, 2009). These brain regions are part of the neurocircuitry of addiction. Earlier findings have shown that blockade of Hcrt-r1 receptors in the PVT did not produce any reduction of cue-induced reinstatement of cocaine seeking (James et al., 2011) suggesting that antagonizing Hcrt-r2 within this brain region may be more efficient in blocking drugs of abuse effects. In agreement with this hypotheses, other studies have shown that microinjection of the Hcrt-r2 antagonist (2*S*)-1-(3,4-dihydro-6,7-dimethoxy-2(1*H*)-isoquinolinyl)-3,3-dimethyl-2-[(4-pyridinylmethyl)amino]-1-butanone hydrochloride (TCSOX229) but not SB334867 into the PVT significantly attenuated the expression of naloxone-induced conditioned place aversion (CPA; Li et al., 2011), implying a specific role for PVT Hcrt-r2 in mediating morphine withdrawal. Furthermore, acute nicotine increased Fos expression in Orx/Hcrt neurons that project from the lateral hypothalamus to the PVT (Pasumarthi and Fadel, 2008), suggesting the participation of this pathway in nicotine arousal. A role for Orx/Hcrt projections from the lateral hypothalamus to the PVT in ethanol seeking is supported by findings that showed that alcohol-related contextual cues activate these neurons (Dayas et al., 2008). Specifically, more Fos-positive hypothalamic Orx/Hcrt neurons were observed in rats exposed to contextual stimuli previously associated with ethanol availability *vs*. rats exposed to the same stimuli previously paired with non-reward, and the ethanol-related stimuli increased the number of Fos-positive PVT neurons that were closely associated with Orx/Hcrt fibers (Dayas et al., 2008).

Importantly, the PVT has been reported to participate in the regulation of feeding. For example, lesions of the PVT (Bhatnagar and Dallman, 1999) or inhibition of PVT neurons with GABA_A_ antagonist muscimol (Stratford and Wirtshafter, 2013) were shown to increase feeding. Likewise, electrolytic lesion of the PVT induced an attenuation of increased locomotion and blood corticosterone levels normally produced by the anticipation to obtain food (Nakahara et al., 2004). Only a few examples of the role of this thalamic nucleus in food intake regulation are mentioned here, and discussing this issue further is beyond the scope of the present review. The following sections discuss recent findings from this laboratory that describe the specific involvement of the PVT (and Orx/Hcrt transmission) in drug-seeking behavior *vs*. normal motivated behavior toward a conventional reinforcer.

## The PVT is differentially recruited by cocaine *vs*. natural reward: correlation with cocaine seeking

Further evidence from this laboratory (Martin-Fardon et al., 2013) has demonstrated a differential recruitment pattern of the PVT by cocaine-related stimuli *vs*. stimuli paired with a highly palatable conventional reinforcer, sweetened condensed milk (SCM). The aim of this study was to establish the recruitment pattern of the PVT induced by presentation of a discriminative stimulus (S^D^) conditioned to cocaine or SCM using an animal model of relapse described earlier (e.g., Baptista et al., 2004; Martin-Fardon et al., 2007, 2009). Briefly, male Wistar rats were trained to associate the S^D^ with the availability of cocaine or SCM (S^+^) *vs*. saline or non-reward (S^−^). Following the extinction of cocaine- and SCM-reinforced responding, the rats were presented with the respective S^+^ or S^−^ alone. Presentation of the cocaine S^+^ or SCM S^+^ (but not the non-reward S^−^) after extinction stimuli elicited identical levels of reinstatement as described in earlier studies (Baptista et al., 2004; Martin-Fardon et al., 2007, 2009). The brains were labeled for Fos in the PVT, and Fos-positive neurons were counted following cocaine S^+^ or SCM S^+^ presentation and compared with counts obtained following S^−^ presentation. Presentation of the cocaine S^+^ but not saline S^−^ activated c-fos. In contrast, presentation of both the SCM S^+^ and non-reward S^−^ produced identical neural activation. A correlation plot between the reinstatement responses and number of Fos-positive cells in the PVT revealed a significant correlation in the cocaine group but not in the SCM group (Martin-Fardon et al., 2013). These data suggest that the PVT is specifically recruited during the conditioned reinstatement of cocaine seeking but not SCM seeking, further supporting the hypothesis that this thalamic structure is involved in the drug addiction circuitry.

## Orx/Hcrt in the pvt mediates cocaine-seeking behavior in rats

The significant correlation in the cocaine group but not in the SCM group strongly suggests that cocaine induces the dysregulation of neurotransmission in the PVT. The aim of the next study was to investigate the specific role of PVT Orx/Hcrt transmission in cocaine seeking *vs*. behavior motivated toward SCM seeking. Male Wistar rats were trained to self-administer short-access cocaine (ShA; 2 h/day), long-access cocaine (LgA; 6 h/day; i.e., an animal model of cocaine dependence), or SCM (30 min/day) for a total of 21 days and then subjected to daily extinction training for 14 days. The following day, the rats received intra-PVT microinjections of Orx-A/Hcrt-1 (0, 0.25, 0.5, 1, and 2 μg) and then placed into operant chambers under extinction conditions for 2 h. Orx-A/Hcrt-1 reinstated ShA and LgA cocaine seeking and SCM seeking but with different dose-response profiles. The effects of Orx-A/Hcrt-1-induced reinstatement on cocaine seeking in the ShA group were characterized by an inverted U-shaped dose-effect function, with low doses but not high doses eliciting reinstatement (Matzeu et al., 2013). In contrast, Orx-A/Hcrt-1 induced reinstatement in the SCM group at high but not low doses. A leftward shift in the Orx-A/Hcrt-1 dose-effect function was observed for the reinstatement of ShA cocaine seeking compared with SCM seeking. Additionally, Orx-A/Hcrt-1-induced reinstatement in the LgA group produced a left-upward shift of the dose-response function compared with the SCM group and an upward shift compared with the ShA group. These findings suggest that a history of cocaine dependence leads to neuroadaptive changes at the level of the PVT, resulting in “sensitization” of LH-PVT-Orx/Hcrt transmission, reflected by increased sensitivity (i.e., a leftward shift) and exacerbated behavioral responses (i.e., an upward shift) to the effects of Orx-A/Hcrt-1, further implicating Orx/Hcrt-PVT transmission in cocaine-seeking behavior and the specific involvement of the PVT in the neurocircuitry associated with cocaine seeking. Knowing that Orx/Hcrt participates in the regulation of a multitude of physiological processes, one may argue that exogenous administration of Orx/Hcrt into the PVT may produce nonspecific side effects. Recently, it was reported that intra-PVT administration of Orx-A at doses 1.5- to 4.5-fold higher than the maximum dose used here significantly increased freezing and grooming behavior, which may interfere with (i.e., reduce) operant responding (Li et al., 2010). However, in the present study, Orx-A administration reinstated (increased) reward-seeking behavior; therefore, over the dose range selected, Orx-A should not have produced any nonspecific changes in “emotional” behavior that could account for the different dose-response functions produced in the different groups.

## Conclusion

A greater understanding of the neurotransmission that underlies compulsive behaviors associated with addiction will provide a more targeted and efficacious means of establishing and prolonging drug and alcohol abstinence. Data from this laboratory and the literature indicate that Orx/Hcrt-PVT transmission plays a distinctive role in behavior motivated by stimuli conditioned to drugs *vs*. natural rewards and that a history of cocaine dependence changes the sensitivity of the PVT to the Orx-A priming effect. This suggests that drugs of abuse in general dysregulate neurotransmission in the PVT and that with long-term drug or alcohol use, the Orx/Hcrt system acquires a preferential role in mediating drug of abuse seeking *vs*. natural reward seeking. What remains to be clarified are the neuromechanisms behind this differential involvement of Orx/Hcrt-PVT transmission. One hypothesis is that a history of protracted drug abuse induces dysregulation of lateral hypothalamus-Orx/Hcrt-PVT neurotransmission, reflected by a change in Orx/Hcrt receptor expression in the PVT or an alteration of Orx/Hcrt production in the lateral hypothalamus that in turn is reflected by a correlation between PVT activation and cocaine-seeking behavior. A history of drug self-administration may also induce neuroadaptations (e.g., enhanced synaptic strength) in the PVT that in turn perturbs its “normal” function toward excessive drug-directed behavior.

Considering the importance of relapse prevention in postdependent individuals, it would be important to determine whether the effects of pharmacological tools (e.g., Hcrt-r antagonists) change in postdependent individuals, as described earlier for metabotropic glutamate receptors (Aujla et al., 2008; Hao et al., 2010; Sidhpura et al., 2010; Kufahl et al., 2011) and the nociceptin system (e.g., Economidou et al., 2008; Martin-Fardon et al., 2010; Aujla et al., 2013) and whether these effects are mediated by the PVT. The literature and data generated by our laboratory strongly support a previously unrecognized mechanism, namely the dysregulation of Orx/Hcrt-PVT transmission, in the etiology of drug dependence, which may help identify novel therapeutic targets for drug addiction.

## Conflict of interest statement

The authors declare that the research was conducted in the absence of any commercial or financial relationships that could be construed as a potential conflict of interest.
